# Importance of Genetic Testing in Dilated Cardiomyopathy: Applications and Challenges in Clinical Practice

**DOI:** 10.5935/abc.20190144

**Published:** 2019-08

**Authors:** Arsonval Lamounier Júnior, Filipe Ferrari, Renato Max, Luiz Eduardo Fonteles Ritt, Ricardo Stein

**Affiliations:** 1 Health in Code S.L., Scientific Department, A Coruña - Spain; 2 Universidade da Coruña, GRINCAR (Cardiovascular Research Group), A Coruña - Spain; 3 Graduate Program in Cardiology and Cardiovascular Sciences, Hospital de Clínicas de Porto Alegre, Universidade Federal do Rio Grande do Sul, Porto Alegre, RS - Brazil; 4 Exercise Cardiology Research Group (CardioEx), Hospital de Clínicas de Porto Alegre, Universidade Federal do Rio Grande do Sul, Porto Alegre, RS - Brazil; 5 Hospital Universitário Onofre Lopes, Natal, RN - Brazil; 6 Escola Bahiana de Medicina e Saúde Pública, Salvador, BA - Brazil; 7 Hospital Cárdio Pulmonar, Salvador, BA - Brazil

**Keywords:** Cardiomyopathy, Dilated/genetics, Ventricular Dysfunction, Left, Heart Failure, Genetic Testing/methods, Heart Transplantation

## Abstract

Dilated cardiomyopathy (DCM) is a clinical syndrome characterized by left ventricular dilatation and contractile dysfunction. It is the most common cause of heart failure in young adults. The advent of next-generation sequencing has contributed to the discovery of a large amount of genomic data related to DCM. Mutations involving genes that encode cytoskeletal proteins, the sarcomere, and ion channels account for approximately 40% of cases previously classified as idiopathic DCM. In this scenario, geneticists and cardiovascular genetics specialists have begun to work together, building knowledge and establishing more accurate diagnoses. However, proper interpretation of genetic results is essential and multidisciplinary teams dedicated to the management and analysis of the obtained information should be considered. In this review, we approach genetic factors associated with DCM and their prognostic relevance and discuss how the use of genetic testing, when well recommended, can help cardiologists in the decision-making process.

## Introduction

Primary cardiomyopathies (PCMs) are a heterogeneous group composed predominantly by genetic diseases associated with pathological alterations of myocardial structure and function.^1-3^ These diseases often progress to heart failure (HF), with dilated cardiomyopathy (DCM) being the main indication for heart transplantation (HTx).^3^ Currently, the prevalence of idiopathic DCM is estimated at around 1 case per 2,500 population, but authors such as Hershberger et al.^4^ describe a frequency ten times greater.^4^ Particularly in the last two decades, a greater understanding on the etiology and clinical course of many of these diseases has been achieved.^5,6^ This has been possible by substantial advances in the use of genetic diagnosis at cardiomyopathy clinics and research centers around the world.

Traditionally, DCM is defined as dilatation of the left ventricle or both ventricles, with consequent impairment in myocardial contractility, in the absence of abnormal overload and/or ischemic heart disease.^4-6^ However, this syndrome can encompass a wide range of genetic and acquired disorders that can be expressed to a greater or lesser impact over the patient’s life course. Some individuals with specific mutations detected in the early DCM-stages may present intermediate phenotypes that do not meet the classical definition of the disease.^2,4^ For this reason, the formulation of some concepts that define subgroups of patients with this syndrome would be relevant, such as the case of hypokinetic non-DCM,^2^ where systolic dysfunction may occur without left ventricular dilatation. In fact, the observed clinical heterogeneity is the partial reflex of the various genes related to sarcomere proteins, cytoskeleton, intercellular connections, the cell membrane, and ion channels (Table 1),^2,4,7,8^ which have been implicated in DCM. Many of these genes have been also associated with other forms of cardiomyopathies (left ventricle non-compaction [LVNC], arrhythmogenic, hypertrophic, and restrictive), and the prevalence of pathogenic variants in each of these genes is distinct for each disorder.^4,7^ Mutations with pathogenic potential are identified in up to 40% of cases described as idiopathic DCM, depending on the cohort.^9,10^ Indeed, it has been suggested that genetic testing would have a higher yield (up to 70%) in cohorts of patients with idiopathic DCM already waitlisted for HTx.^11,12^

**Table 1:** Main genes associated with dilated cardiomyopathy

Gene	Protein	Estimated contribution	Association with other cardiomyopathies	Other phenotypes	Inheritance	Level of evidence*
**Sarcomere**						
TTN	Titin	18-25%	LVNC	Myopathies	AD	1
TNNT2	Troponin T type 2	2-3%	HCM, LVNC	-	AD	1
TNNI3	Troponin I3, cardiac type	1-2%	HCM, RCM	-	AR	1
TPM1	Tropomyosin 1	1-2%	HCM, LVNC	Congenital heart disease	AD	1
MYH7	Miosina-7 (beta-myosin heavy chain)	3-5%	HCM, LVNC	Myopathies	AD	1
MYBPC3	Myosin-binding protein C	2%	HCM, LVNC	-	AD	1
BAG3	Bcl-2-associated athanogene 3	2%	-	Myofibrillar myopathy	AD	1
ACTC1	Actin alpha cardiac muscle 1	<1%	HCM, LVNC	-	AD	1
**Cytoskeleton**						
ACTN2	Actin alpha cardiac muscle 2	< 1%	HCM	Congenital heart disease	AD	2
FLNC	Filamin C	2.2%	HCM, RCM	-		1
LDB3	LIM domain binding 3	<1%	NA	Myofibrillar myopathy	AD	2
ANKRD1	Ankyrin repeat domain 1	< 1%	HCM	Congenital heart disease	AD	3
VCL	Vinculin	1%	NA	-	AD	3
JUP	Junction plakoglobin	1 %	ACM	Naxos disease	AD/AR	1
DMD	Dystrophin	1%	NA	Duchenne and Becker muscular dystrophy	X-linked	1
DES	Desmin	1-2%	HCM, RCM	Myofibrillar myopathy	AD	1
**Cell Membrane**						
LMNA	Lamin A/C	5-10%	HCM	Muscle myopathies, lipodystrophies, progeria	AD	1
EMD	Emerin	NA	ACM	Emery-Dreifuss muscular dystrophy	X-linked	1
Ion Channels						
SCN5A	Sodium voltage-gated channel alpha subunit 5	2-3%	LVNC	Brugada syndrome/LQTS	AD	1
ABCC9	ATP binding cassette subfamily C member 9	< 1%	-	Osteochondrodysplasia	AD	3
**Desmosome**						
DSC2	Desmoscollin-2	1-2%	ACM	Palmoplantar keratoderma	AD	1
DSG2	Desmoglein 2	1-2%	ACM	-	AD/digenic	1
DSP	Desmoplakin	3%	ACM	Carvajal syndrome	AR	1
PKP2	Plakophilin 2	<5%	ACM	-	AD	1
**Lysosome**						
LAMP2	Lysosome-associated membrane protein 2	4%	HCM	Danon disease	X-linked	1
**Sarcoplasmic** **Reticulum**						
PNL	Phospholamban	1%	HCM, ACM	-	AD	1
RYR2	Ryanodine receptor 2	NA	-	CPVT	AD	2
RBM20	RNA binding motif protein 20	2%	LVNC	-	AD	1

HCM: hypertrophic cardiomyopathy; RCM: restrictive cardiomyopathy; LVNC: Left ventricle non-compaction cardiomyopathy; ACM: arrhythmogenic cardiomyopathy; CPVT: catecholaminergic polymorphic ventricular tachycardia; LQTS: long QT syndrome; NA: not available; AD: autosomal dominant; AR: autosomal recessive.

* The genes were classified according to three levels.

Level 1: Multiple studies, variants and families reported and cosegregation with established disease. Level 2: Single or few studies, variants and families reported and limited cosegregation observed. Level 3: Single or few studies, variants and families reported and unestablished cosegregation.

In the last decade, recommendations for the use of genetic testing in familial DCM have been established by respective guidelines.^9^ Genetic testing can help in the management of patients and their relatives, as well as optimize the risk stratification. Careful clinical evaluation, a thorough family history, and the results of genetic testing are the cornerstones of this approach. Considering the incipient use of genetics in DCM management in Brazil, the aim of this review is to present and discuss the importance of molecular testing in the DCM spectrum.

### Methods for genetic diagnosis in dilated cardiomyopathy

The use of next-generation sequencing (NGS) platforms has enabled two approaches for the genetic diagnosis of DCM:

1. Whole-exome sequencing: this approach covers “all” exons and flanking regions of the human genome (in practice, it considers only those genes for which correlated clinical information already exists). Exome sequencing has been more used in applied clinical research, resulting in the discovery of new genes potentially associated with DCM.^13,14^ These “new” genes are also called candidate genes because the level of evidence supporting its pathogenic potential is still low or uncertain. It would result in a greater number of unknown clinical significance variants;
2. Targeted NGS: NGS panels sequence a certain number of genes for which there is higher evidence of a causal association with DCM (Table 1).^15,16^ The large volume of cases already described in carriers of pathogenic/likely pathogenic variants in these genes raised useful information for clinical decision-making;^17^ it is important to emphasize that most of the mutations reported in DCM are exclusive to a single family, which leads to barriers in the interpretation of the genetic data. Therefore, integration of clinical manifestations and family history is essential in the decision-making process.^17^ There are around a hundred genes associated with DCM, with different levels of evidence for their associations. Furthermore, it bears stressing that the presence of a genetic mutation does not always mean that the disease will develop.^18^ The best-documented genes are listed in Table 1.

In recent years, the increasingly widespread use of NGS panels has allowed the identification of a significant number of individuals with variants in the same gene. This population has been enabling relevant clinical descriptions in DCM, as in the most recent studies addressing different genes (*TTN*, *LMNA*, *FLNC*, or *BAG3*).^19-21^ Cosegregation of variants in these genes has been demonstrated as disease-causing in multiple DCM-families, and the ever-increasing number of identified carriers has enabled genotype-phenotype correlation analyses on the prognosis of the disease.^22,23^

Briefly, genetic testing through the aforementioned techniques can help cardiologists in three important clinical scenarios:^8-10,24,25^

1. familial management;
2. etiological definition and;
3. assertive risk stratification.

### Importance of family screening in dilated cardiomyopathy

A cohort study with advanced HF patients waiting in the transplant list shows that the diagnosis of familial DCM (FDCM) was systematically neglected.^26^ The mere use of the pedigree tool increased the prevalence of this diagnosis from 4.1%to 26%. Prospective cohorts have since found that 25 to 40% of non-ischemic DCM cases are in fact familial,^9,24^ drawing attention to the hereditary component of this syndrome, as well as to the importance of early detection and treatment of affected relatives. In this context, pedigree with at least three generations and the use of genetic testing is strongly recommended.^9^

The identification of a pathogenic or a likely pathogenic variant in an index case (proband) allows the entire family of the patient may benefit from genetic screening.^4,9^ This is particularly useful in cases where clinical evaluation alone has not been able to establish the diagnosis in a relative.^2,4^ In addition, the early identification of a family member carrier who is asymptomatic, or in the subclinical phase of the disease, may be particularly relevant when genetic testing reveals etiologies with greater arrhythmogenic potential or known to evolve faster.^19,20^ Moreover, in this scenario, it would be possible to apply measures to delay disease progression or even avert a fulminant outcome. On the other hand, regular follow-up of the index case's relatives that are identified as non-carriers of a pathogenic variant is not recommended;^9^ this avoids unnecessary health expenditures and prevents additional psychological stress to the patient/family.

### Etiological definition and associated prognosis

**TTN truncating variants**: a milestone in knowledge about the role of genetics in DCM was a study by Herman et al.^11^ In this publication, titin-truncating variants (TTNtv) were identified in 25% of cases of FDCM and in 18% of sporadic DCM-cases. These prevalence were obtained across three different cohorts, with a frequency of TTNtv between 8 and 40%. It is worth noting that higher frequencies were observed in subjects undergoing HTx or with severe systolic dysfunction. Since then, other studies have sought to ascertain the natural history of DCM by assessing TTNtv patients.^24,27,28^ No difference was observed in the incidence of outcomes among affected carriers vs. non-carriers,^27^ as there was no difference in mesocardial fibrosis between these groups.^28^ However, males with TTNtv manifested the disease at younger ages than female carries (78% vs. 30% of women at age 40).^27^ Other authors have identified that affected TTNtv carriers would have an earlier outcome event (death from any cause, waiting for HTx, or requiring a ventricular-assist device) than non-carriers.^28^ In this same line, male carriers had a lower survival rate (28% of men had a cardiovascular event vs. 8% of women before age 50), considering the outcomes death by HF, HTx, or use of a ventricular assist device.^27^

In a robust sample (n = 558), a high incidence of cardiovascular death starting at age 40 years was observed in patients with TTNtv.^29^ In this cohort, the incidence of cardiovascular events was again higher in men than in women “(1.25% vs. 0.75%/year between the ages of 40-60), with sudden cardiac death being the most frequent outcome. In addition, several publications have described a high incidence of atrial fibrillation, as well as sustained and non-sustained ventricular tachycardia, in these patients.^24,25,28,30^

Although according to the literature, the presence of TTNtv is associated with early onset of arrhythmic manifestations, it has not yet been possible to define a characteristic phenotype for these patients, unlike for patients with mutations in other DCM-related genes. Moreover, it has been proposed that TTNtv may be acting as a susceptibility genetic substrate to different DCM-types (anthracycline-induced, peripartum, and alcoholic).^27-29^ Finally, patients with titin cardiomyopathy appear to have a more favorable clinical course and respond better to drug therapy than those affected by variants in the lamin (*LMNA*) gene.^31-33^

**Lamin**: *LMNA* pathogenic variants produce a well-characterized DCM phenotype associated with conduction disease/malignant arrhythmias, also called cardiolaminopathies.^34^ Phenotypic expression have been described as a progressive atrioventricular conduction disease which usually precedes ventricular dysfunction and/or ventricular arrhythmias, although ventricular or supraventricular arrhythmias (especially atrial fibrillation) may be identified as the first manifestation.^19,34^ Diagnosis is usually established after age 20, with high penetrance (>90%) after 40 years.^35^ Once the first symptoms have manifested, cardiolaminopathies can progress to advanced HF faster than primary DCM of other etiologies.^19,36^*LMNA* mutations prevalence ranges from 5-10% in FDCM cohorts and 2-5% in sporadic cases, accounting for up to 30% of the DCM-cases associated with conduction disease/arrhythmias. Their prevalence is lower in cases of isolated DCM (non-arrhythmic).^34,35^

Between 2013 and 2015, Hasselberg et al.^37^ identified a 6% prevalence of *LMNA* gene mutations in a cohort of 79 Norwegians young with FDCM. A particularly high proportion of carriers (19%) required HTx. In another study, 122 affected *LMNA* carriers were followed for seven years; 27 progressed to terminal DCM or death. It is worth noting that, in this same cohort, asymptomatic carriers had a 9% annual incidence of any documented cardiac event over 4 years of follow-up.^19^ These findings suggest a significant unfavorable prognosis and faster progression to HTx/death than in DCMs with other etiologies.

These clinical and epidemiological profile supports the recommendations for genetic testing of patients with non-ischemic DCM. Furthermore, an early implant of a cardioverter/defibrillator (ICD) should be considered when a *LMNA* pathogenic variant is identified (Class of recommendation IIa, level of evidence B) in a patient with risk factors.^38^ Based on the study published in 2012 that enrolled 269 patients with cardiolaminopathy, the risk of cardiac events is highest when the patient has two or more of these risk criteria at the time of diagnosis: 1) non-sustained ventricular tachycardia; 2) left ventricular ejection fraction <45%; 3) male sex; and 4) a truncating-type variant.^39^ Since then, other authors have proposed that not only truncating variants but also missense-type genetic variants, would be relevant due to the potential for sudden death.^40^ Finally, a new risk score for patients with DCM associated with *LMNA* variants should be published soon. We hope that it will increase the accuracy of risk stratification in these patients.

**Other genes**: Some sarcomere genes more often associated with hypertrophic cardiomyopathy (HCM) may also cause DCM.^25^ The beta-myosin heavy chain (*MYH7*), troponin T (*TNNT2*), and tropomyosin (*TPM1*) sarcomere genes are those for which associated prognostic information is available. Depending on the location of the genetic variant in the *MYH7* gene, the natural history may be particularly severe, similarly to that observed occurs in cases of DCM caused by *TNNT2* mutations.^25^*TPM1* variants cause less than 1% of DCM cases but account for a significant portion of pediatric forms of this disease, in which rapid progression to death or HTx is not uncommon.^25^ A meta-analysis of nearly 8,100 patients evaluated genotype-phenotype correlations of DCM with the *TTN* and *LMNA* genes, as well as genes encoding sarcomere proteins such as myosin-binding protein C (*MYBPC3*), *MYH7*, *TNNT2*, troponin I (*TNNI3*), RNA-binding protein 20 (*RBM20*), and phospholamban (*PLN*).^41^ A significantly higher frequency of HTx was observed in patients with *LMNA* mutations (27%) than for carriers of *RBM20* and *MYBPC3*mutations (~10% each). Across the different genes examined, those affected were predominantly male (79% for *MYBPC3* mutations and 69% for *LMNA* and *MYH7* variants), except for *PLN* mutations (46% men).

More recently, the filamin C (*FLNC*) and Bcl-2-associated athanogene 3 (*BAG3*) genes have had their role in the DCM natural history characterized in high-impact publications.^20,21,42^*FLNC* was associated with a more arrhythmogenic profile, and *BAG3*, with a greater number of HF-related events. *FLNC* truncating variants were found cosegregating in 28 affected families with a particular form of arrhythmogenic/DCM.^20^ The most prevalent clinical features were ventricular dilatation (68%), systolic dysfunction (46%), and myocardial fibrosis (67%). Ventricular arrhythmias were documented in 82% of the patients, and cases of sudden death were reported in 21/28 families. Another study identified *FLNC* truncation variants in 2.2% of DCM-patients, 85% of whom had ventricular arrhythmias and/or sudden death.^42^ Additional right-heart involvement was reported in 38% of the cases. Thus, early ICD implantation could be considered in these patients, even when they do not meet the criteria established in current DCM guidelines.^9,20^

In recent years, several isolated BAG3 reports have been described in DCM-families.^43-47^ In a meaningful way, BAG3 associated phenotype was recently defined with data from a cohort of 129 carriers.^19^ After a mean follow-up of 38 months, the number of carriers affected with DCM raised from 57% to 68%. It represents 26% of the carriers who initially had a negative phenotype but manifested the disease. Considering carriers over the age of 40, 80% were phenotype-positive. In this sample, the incidence of cardiac events in carriers of *BAG3* variants with DCM was 5.1% per year (outcomes: sustained ventricular tachycardia, sudden death, HF death, need for ventricular assist device and HTx), with a predominance of HF-events versus a lower number of arrhythmic outcomes. Male patients, those with systolic dysfunction, or increased ventricular diameter had the highest incidence of events during the follow-up.^21^ Based on these findings, variants of the *BAG3* gene do not appear to be related to a need for early ICD implantation, unlike *FLNC* mutations.

### Genetic heterogeneity and overlapping phenotype

Many of the genes described as disease-causing in patients with DCM are also associated with the development of other forms of PCMs (Table 1). This fact may result in the presence of more than one phenotype in the same pedigree or overlapping phenotypes in the same individual, as occurs, for example, with LVNC and DCM.^48,49^

In a cohort of 95 patients with LVNC (68 unrelated individuals and 27 relatives, 23% of cases familial), a genetic variant was identified in 38% of the cases.^49^ The most frequent genes were *TTN*, *LMNA*, and *MYBPC3*; one family was affected by a *RBM20* mutation. In this family, three generations were affected on the maternal side, and the index case underwent to HTx at the age of 21 (3 years after diagnosis). The number of major cardiovascular events in this cohort was significantly higher in patients with LVNC than those probands with non-ischemic DCM of known etiology. Approximately 10% of the patients with LVNC required HTx vs. 2.8% in the non-ischemic DCM group.^49^ One of the families with LVNC in this cohort was found carrying a *MYH7* gene variant which has been associated in different studies with malignant-HCM. Although the affected individuals did not meet definitive criteria for HCM, several had an appreciable increase in myocardial wall thickness.^49^ This should draw the attention of cardiologists to the phenotypic heterogeneity of PCMs, as well as to the need to bear in mind what an etiological diagnosis can represent in terms of the natural history of the disease.

Genes usually related to arrhythmogenic cardiomyopathy (ACM) may produce indistinguishable clinical phenotype from those with DCM. Patients affected by desmoplakin (*DSP*) or *FLNC* truncating variants, for instance, may be affected by a form of ACM with exclusive left ventricle involvement.^20,50,51^ There are several reports of DCM-patients affected by mutations in desmosomal genes that do not fulfil any (or only some) of the arrhythmogenic right ventricular dysplasia diagnostic criteria.^50-52^

In a study of 89 unrelated end-stage DCM patients requiring HTx, screening of the five most common desmosomal genes (*PKP2*, *DSP*, *JUP*, *DSC2*, *DSG2*) identified genetic variants in 18% of the probands.^51^ Genetic testing in relatives identified additionally 38 carriers, including some with subclinical DCM. Histopathological analysis of explanted hearts was heterogeneous; some cases showed fibro-fatty infiltration of the right ventricle, some of the left ventricle, and some had no observable fibro-fatty replacement.^52^

### Final considerations

In view of the foregoing, we conclude that the PCMs, particularly the dilated form (DCM), reflect a complex, highly heterogeneous syndrome of challenging diagnosis, prognosis, and treatment. In this scenario, molecular NGS diagnosis can be a very useful tool for clinical cardiologists practice. Although still incipient in Brazil, the use of genetic testing in DCM and HTx services should be considered, since etiological diagnosis often allows a more assertive clinical management and risk stratification. Moreover, clinical and genetic screening of patients' relatives with these conditions is somewhat neglected, although it is a recommended approach. In this way, cardiomyopathy units and HTx services, as well as correlated research groups, should address this topic more incisively and focus on disseminating knowledge among health professionals and in the society as a whole.

Despite its potential benefits, the limitations of genetic testing should not be overlooked. The possible psychological impact of the genetic testing results on patients and their families should be anticipated and discussed. In addition, genetic testing cannot determine whether the proband will develop symptoms, as well as the severity of these potential symptoms.^53^

Finally, we illustrate the present article with a clinical case from our practice in which genetic testing was part of the clinical evaluation.

**Figure 1:**
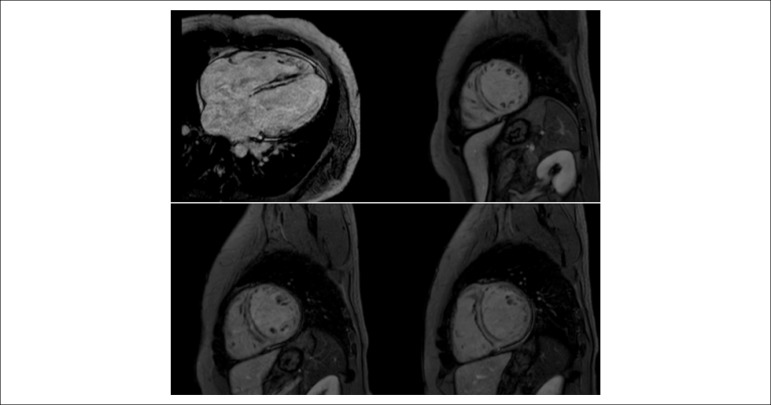
Cardiac magnetic resonance imaging showing extensive and diffuse area of mesocardial fibrosis.

**Figure 2:**
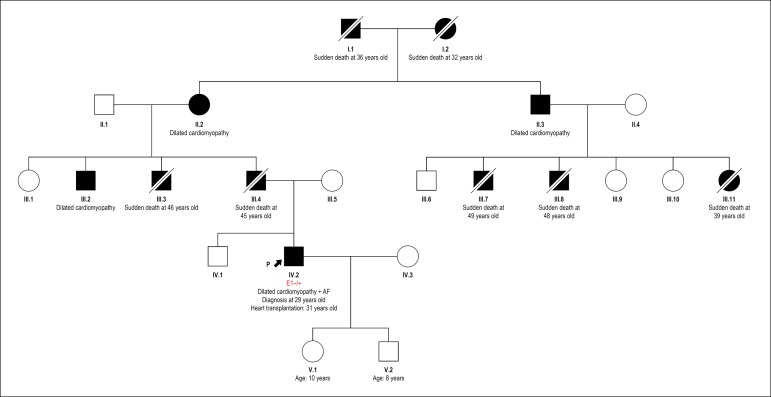
Index case heredogram showing involvement in first, second and third degree relatives. AF: atrial fibrillation.

**Clinical case:** A 30-year-old male was hospitalized due to congestive HF (NYHA functional class III). After 1 year of occasional palpitations, a diagnosis of DCM was established. Cardiac magnetic resonance imaging, which revealed diffuse hypokinesis, left ventricular dysfunction (left ventricular ejection fraction, 46%), and diffuse mesoepicardial fibrosis (Figure 1). Comparison with a previous echocardiogram performed two years latter suggested a rapid clinical progression (left ventricular ejection fraction, 32%); systolic and diastolic diameters, 49 and 58 mm, respectively; left atrial diameter, 44 mm. Echocardiogram showed atrial fibrillation and the coronary angiography did not reveal any evidence of coronary heart disease. Taking into consideration, the severity of the case and a positive family history of sudden death (Figure 2), genetic testing was performed (NGS panel for DCM). A *LMNA* p.Leu176Pro variant was identified, providing etiological confirmation for familial DCM. Based on these findings an ICD was implanted for primary prevention, despite of the left ventricular ejection fraction > 30%. Consistent with descriptions in the literature, cardiolaminopathy of the patient did not respond adequately to optimized clinical treatment and progressed rapidly to HTx. One year later, the patient developed anasarca and was hospitalized again, requiring positive inotropic supporting with intravenous agents. HTx was performed 2 months later. The patient’s children (both under 10 years of age) were apparently healthy. As the mean age of diagnosis of *LMNA* gene carriers is from the third decade of life onward, ethical recommendations for genetic testing in underage relatives were followed, respecting the appropriate age for genetic counseling.^54^ However, it is important to mention that cases of children affected by *LMNA* cardiomyopathy have been reported rarely, which should call into question the current expectant management of young children of patients with cardiolaminopathies.^55^
